# A New Azhdarchid Pterosaur from the Late Cretaceous of the Transylvanian Basin, Romania: Implications for Azhdarchid Diversity and Distribution

**DOI:** 10.1371/journal.pone.0054268

**Published:** 2013-01-30

**Authors:** Mátyás Vremir, Alexander W. A. Kellner, Darren Naish, Gareth J. Dyke

**Affiliations:** 1 Department of Natural Sciences, Transylvanian Museum Society, Cluj-Napoca, Romania; 2 Laboratory of Systematics and Taphonomy of Fossil Vertebrates, Department of Geology and Paleontology, Museu Nacional/Universidade Federal do Rio de Janeiro, Rio de Janeiro, São Cristóvão, Brazil; 3 Ocean and Earth Sciences, University of Southampton, Southampton, United Kingdom; 4 Institute of Life Sciences, University of Southampton, Southampton, United Kingdom; Team ‘Evo-Devo of Vertebrate Dentition’, France

## Abstract

We describe a new taxon of medium-sized (wing span ca. 3 m) azhdarchid pterosaur from the Upper Cretaceous Transylvanian Basin (Sebeş Formation) of Romania. This specimen is the most complete European azhdarchid yet reported, comprising a partially articulated series of vertebrae and associated forelimb bones. The new taxon is most similar to the Central Asian *Azhdarcho lancicollis* Nessov but possesses a suite of autapomorphies in its vertebrae that include the relative proportions of cervicals three and four and the presence of elongated prezygapophyseal pedicles. The new taxon is interesting in that it lived contemporaneously with gigantic forms, comparable in size to the famous Romanian *Hatzegopteryx thambema*. The presence of two distinct azhdarchid size classes in a continental depositional environment further strengthens suggestions that these pterosaurs were strongly linked to terrestrial floodplain and wooded environments. To support this discussion, we outline the geological context and taphonomy of our new specimen and place it in context with other known records for this widespread and important Late Cretaceous pterosaurian lineage.

## Introduction

**Figure 1:**
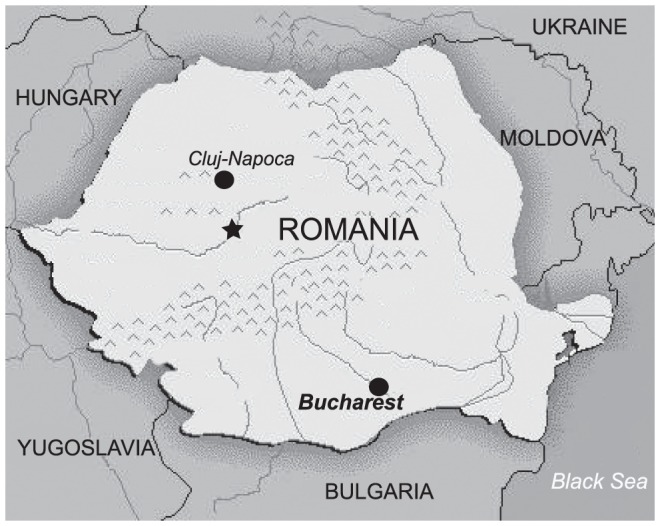
Map of Romania (including the Transylvanian region) showing field area (star).

A rich and phylogenetically diverse latest Cretaceous (Maastrichtian) vertebrate assemblage is now known from Romania. Pterosaur remains are among this assemblage, but remain rare, perhaps due to the poor preservation potential of their bones and other taphonomic factors. Only a handful have so far been collected and described from this region of Eastern Europe: the vast majority are small and from the famous Haţeg Basin in Transylvania [1] (Figure 1).

The first report of presumed pterosaur remains from present-day Romania was a brief mention of three small vertebrae (a possible notarium) collected by Gyula Halaváts in the early 1890's from the Eastern Haţeg Basin and preliminarily identified by Gustav von Arthaber [2]. Some years later, a few small-to-medium-sized “ornithocheirid-like” pterosaur remains (a notarium, several teeth and some unidentified “hollow bones”) were reported from the Maastrichtian of Sînpetru in the Haţeg Basin [3], but Halaváts's material was not discussed further. Much later, additional pterosaur fossils (partial notarium, femur and humerus), collected from the same locality, were identified as belonging to small pteranodontids [4]. Because these specimens were never figured or described in detail (see [5]), their affinities remain uncertain. The “ornithocheirid” notarium from Sînpetru mentioned by Nopcsa [3] was later relocated and redescribed as the sacrum of a maniraptoran theropod [6], [7]. Finally, several ornithocheirid-like teeth have recently been reported from the Haţeg Basin [8], although some may actually belong to small sauropod dinosaurs [1].

Some Romanian pterosaur fossils are anything but small. Most famously, several bones from Haţeg, referred to the giant azhdarchid *Hatzegopteryx thambema* (wingspan: 10–11 m) [9], [10] are known from the Lower or lower Upper Maastrichtian Ciula-Densuş Formation at Vălioara. This taxon was initially described on the basis of an occipital region of a skull (first considered a theropod dinosaur by Weishampel et al. [11]), a posterior palatal fragment and a fragmentary proximal humerus [9]. A fourth specimen, a large femoral shaft (restored length ca. 40 cm), was later described from the Tuştea dinosaur nesting site [12] and is thought to belong to another large individual (with a smaller wingspan: 5–6 m) [1], [12]. Recently, a fifth fragmentary specimen tentatively assigned to *Hatzegopteryx*(?) and also collected at Vălioara was re-located in the paleontological collections of Bucharest University. This fossil was identified by Vremir et al. [1] as a fragmentary anterior portion of a mandibular symphysis and it also comes from a very large individual. Six recently collected Haţeg pterosaur specimens include an incomplete medium-sized distal wing-phalanx (?wing phalanx three) from Boiţa, an incomplete, unfused scapulocoracoid, and a cervical corpus, both from large animals (wingspan: 4.5–5.0 m) and both from Vadu (this latter element may belong to a smaller specimen of *Hatzegopteryx*), and a medium-sized (wingspan: ∼3 m) indeterminate pterodactyloid scapula, unassociated medio-posterior cervical vertebra and a partial humerus (?) from coeval deposits in the Bărbat Valley (Pui locality) [1].

**Figure 2:**
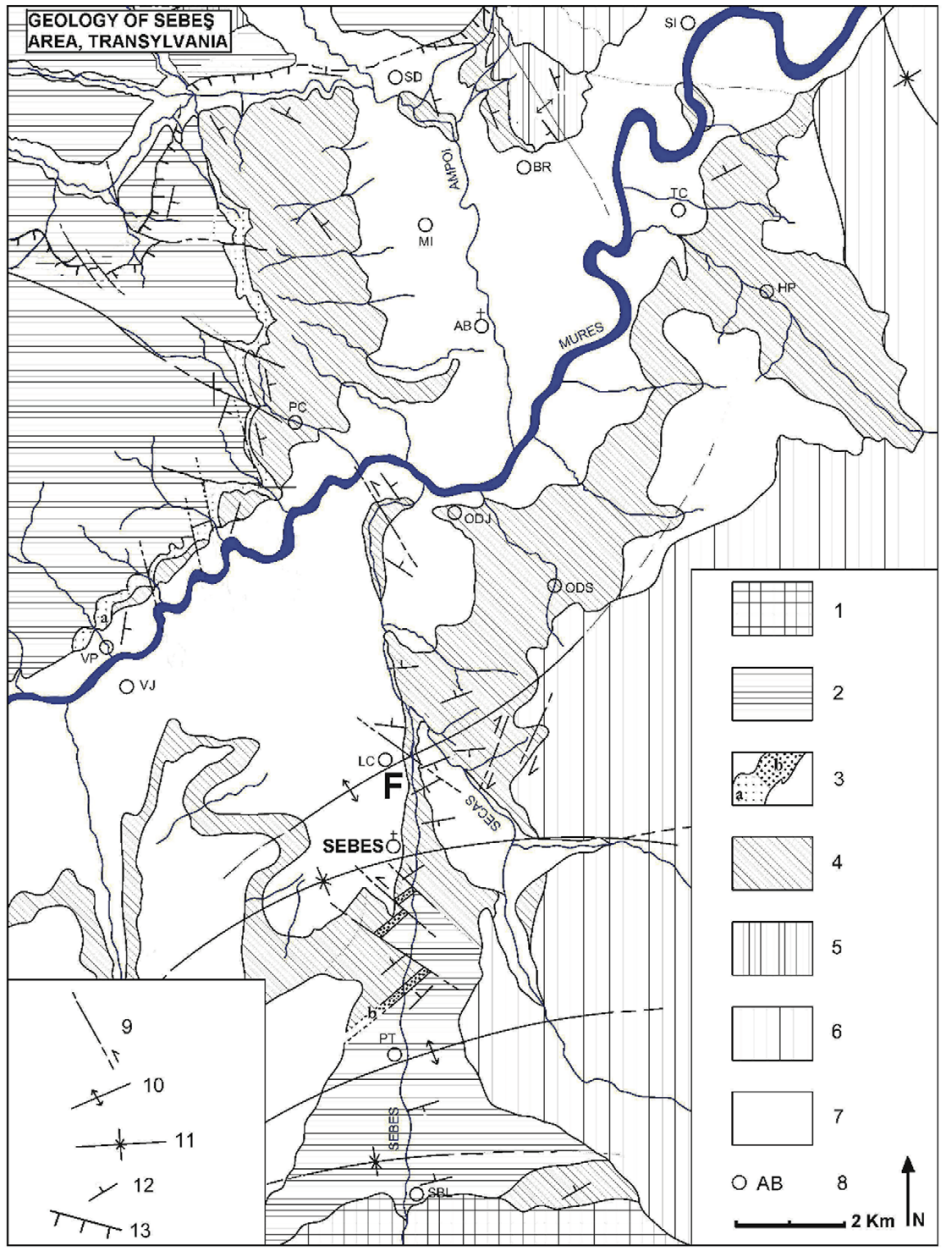
Geological map of the Sebes area. This map is re-drawn from [13], updated and modified: F- SbG/B vertebrate site -the type locality; 1- metamorphic basement (Getic-Supragetic nappe system); 2- Upper Cretaceous (Santonian-Latest Campanian) marine deposits referred to the Bozeş Formation in the base with locally developed coal-bearing fluvial-deltaic facieses referred to “Sebeşel strata”; 3- Transitional deltaic (a) or estuarian-paludal (b) facieses marking the top Bozeş Formation; 4- Upper Cretaceous (Latest Campanian-Maastrichtian/Paleocene?) continental units referred to the Sebeş or Şard (in part) Formations; 5- Undifferentiated Paleogene (Eocene-Oligocene) units referred to Şard (in part), Ighiu and Bărăbanţ Formations; 6- Undifferentiated Miocene and Pliocene marine units referred to the Sîntimbru, Dej and Lopadea Formations; 7- Quaternary cover; 8- Settlements; 9- Faults; 10- Anticlines; 11- Synclines; 12- Dip and strike; 13- Nappe Front.

Another currently less well-known Romanian sedimentary basin of Maastrichtian-age – the Transylvanian Basin – is located to the northeast of Haţeg (Figures 1, 2). To date, only a handful of pterosaur fossils, all discovered in the vicinity of Sebeş City, have been found in this basin [1], [13], [14]. They include a heavily crushed fragment of a wing phalanx (belonging to a very large individual) collected from latest Campanian-lowermost Maastrichtian fluvio-paludal facies (the top of the Bozeş Formation) at the Petreşti-Arini locality [15] and a medium-sized (wingspan: ∼3 m) partial skeleton of an azhdarchid collected from the mid-to-low part of the Sebeş Formation (Sebeş-Glod; Early to Early? Maastrichtian). Giant (wingspans >10 m) pterodactyloid pterosaur remains are also known from the upper part of the Sebeş Formation (likely Late Maastrichtian) and include a huge cervical vertebra [1], [13], [14], a partial coracoid and a proximal syncarpal, all from the Râpa Roşie (Red Cliff) locality.

In this paper, we augment the known pterosaur record from the Romanian Cretaceous by describing the associated remains of the medium-sized azhdarchid from Sebeş-Glod (noted above), the type locality of the exceptionally well-preserved dromaeosaurid theropod *Balaur bondoc* [16], [17]. This fossil material was found and excavated by MV in 2009; subsequently, most of the material was donated by MV to the paleontological collections of the Transylvanian Museum Society in Cluj-Napoca, Romania (EME). We obtained permission from the Transylvanian Museum Society to access their collections and to research this fossil material, a specimen comprising 15 skeletal elements. Some (the anterior-most preserved cervical element and some fragmentary unidentified bones; see below) were collected later and are separately deposited at Babes-Bolyai University, Cluj-Napoca, Romania (UBB). This specimen is the most complete European Maastrichtian azhdarchid found to date and provides critical new information on the morphology, and stratigraphic and geographic distribution, of European azhdarchid pterosaurs.

## Results

### Regional Geological Setting and the Sebeş Formation

**Figure 3:**
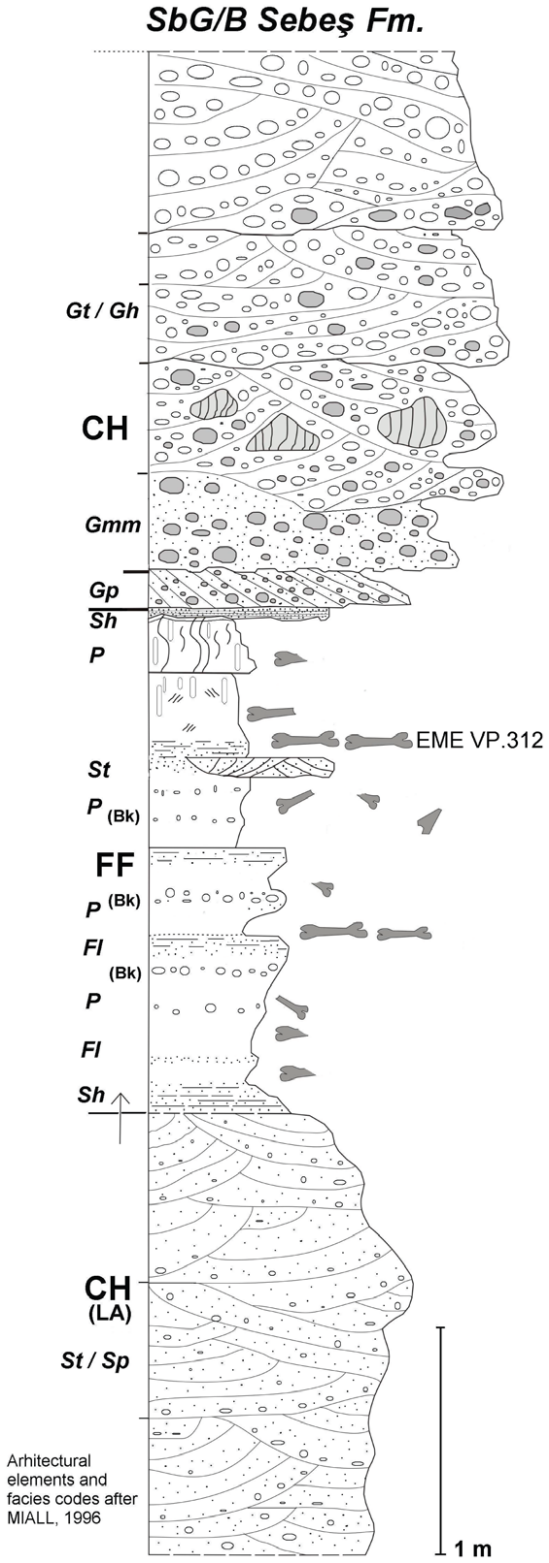
Stratigraphic log of the Sebes Glod (SbG/B) vertebrate site. Facies codes and architectural elements after [64].

Alongside the well-known Upper Cretaceous (Maastrichtian) dinosaur-bearing continental deposits of the Haţeg Basin [18], another set of important Maastrichtian vertebrate sites are located in the Sebeş-Alba region of Romania, about 50 km northeast of Haţeg (Figure 1). Here, Upper Cretaceous sediments form a large-scale transgressive–regressive cycle involving deep-to-shallow marine deposits and continental red beds of Campanian-Maastrichtian age, related to the Laramidian tectonic event [19], [20]. From a stratigraphic point-of-view, the Upper Cretaceous-Paleogene continental deposits in the Sebeş area are represented by several formations that belong to two main tectonostratigraphic megasequences, covering the Santonian–Rupelian interval [13], [21], [22]. The Maastrichtian vertebrate-bearing continental deposits here have been referred either to the recently re-dated Sebeş Formation (SBF) [13], [14], well exposed on the left flank of the Mureş passageway (Secaş Plateau and the Sebeş Valley), or to the Vurpăr (VPF) and Şard (SDF) formations that outcrop on the right flank of the Mureş Valley in the Munceii Vinţului (VPF) and Bilag (SDF) foothills (Figure 2). The whole succession in this region is characterised by lacustrine muds, medium-to-coarse channel associations, and extensive floodplain and overbank facies [13], [23] (Figure 3). The SBF comprises a thick succession of alluvial deposits of dark bluish or gray mudstone, red, dark-brown or yellowish-brown claystone, red silty-clays, and medium-to-coarse poorly-sorted conglomerates with reddish or grayish mostly cross-laminated sandstone interbeds (Figure 3). Deposition took place under various conditions, from proximal alluvial fans to the medium and distal facies of meandering, occasionally braided, fluvial systems that exhibit local evidence for well-developed lacustrine, forrested-swampy, short evaporitic stages and extensive pedogenized floodplain deposits. The thickness of the SBF is approximately 450 m between Sebeş and Râpa Roşie (Red Cliff) the historically designated type section [13], [21] and is unconformably overlapped by transgrading middle Miocene (lower Badenian) marine deposits.

### Local Geological Setting: Localities at Sebeş-Glod

Sebeş-Glod (here coded SbG/A-D) is comprised by suite of restricted outcrops that are located from 1.5 to 3 km north of Sebeş City in Alba County, Romania (Figure 2). All these sites are downstream, alongside, and often in, the Sebeş River and have been briefly discussed by Vremir [13], Csiki et al. [16], Codrea et al. [23] and Brusatte et al. [17]. Here, a roughly 50 m thick profile from the mid-lower section of the SBF, probably late Early Maastrichtian in age, outcrops about 300–350 metres below a major middle-Miocene (Badenian) unconformity that caps the SBF at the Râpa Roşie type section. This profile is also approximately 100 m above the top of the conformably underlying Bozeş Formation (upper Campanian– ?lowermost Maastrichtian) that is well-exposed in the Petreşti-Arini section [15]. The succession here is dominated by coarse, mainly cross-bedded channel associations (i.e., gravel, sandy gravel and cross-laminated sandstones) that are related to a high sinuosity fluvial system, with occasional interbeds of finer-grained red or brownish-red overbank and floodplain associations (Figure 3).

The SbG/B site (from were EME VP 312 was collected) is a small, waterlogged riverbank outcrop, slightly eastward dipping and 6.5 m thick in profile (Figure 3). The basal channel association here is comprised of upward-fining, medium-to-coarse, occasionally pebbly, light pinkish-gray sandstones, grouped in several meters thick progressively thinner trough cross laminated sets that show internal truncation (St/Sp). These sediment associations characterise a lateral accretional facies (LA). Progressive abandonment and lateral migration of the channel is marked by fine, horizontally laminated sands-silts-clays with occasional scour-fill features and flute-marks. These indicate minor crevasses (Sh/Fl), grading into reddish sandy-silty claystone and a mudstone-dominated proximal floodplain association (OF). Fossiliferous layers are grouped into a 2.2 m thick dark-red calcareous silty-claystone sequence that has thinly-laminated and lenticular sandy interbeddings (crevasses), related to periodic flooding events, and several pedogenic calcitic horizons (Bk) that are marked by sparse and occasionally grouped calcareous nodules. Bone fragments and chips are widespread throughout the whole overbank sequence: however, more complete elements of closely associated and/or articulated skeletal parts are to be found immediately above the sandy crevasses, covered by fine sediments. The top of the overbank facies is represented by massive dark-red mudstone, showing abundant tubular burrowings and rhysolites, marking the incipient topsoil horizon. A several-metres-thick, poorly sorted, massive, plannar or concoid cross-laminated multistoried conglomerate channel-fill complex (Gmm, Gp/Gh) covers the whole sequence, bounded by a 4^th^ order flat lower surface. In the basal lag deposits, large cobble-size, reddish sub-angular claystone rip-up clasts are common. The multistory complex CH fills, dominated upward by truncated cross-bedded sets (Gh), may indicate mobile, broad and shallow channels. In such circumstances, a more unstable and dynamic environment, with frequent reactivations and flow direction changes can be reconstructed.

A relatively large number of vertebrate fossils are so far known from the SbG/B site and these originate mainly from the red overbank deposits. Most are fragmentary, isolated bones but more complete elements, closely associated and partially articulated skeletons have also been collected. Upstream from this site, in minor channel deposits associated to OF facies (SbG/C and D sites), mostly fragmentary bones of turtles, crocodylomorphs and sauropod dinosaurs, as well as numerous plant remains, are common. The fauna from here is so far known to comprise various ornithopod (*Zalmoxes*, *Telmatosaurus*), sauropod (*Magyarosaurus*; Titanosauria indet.) and theropod (*Balaur*) dinosaurs, abundant stem-turtles (*Kallokibotion*) and two crocodylomorphs alongside rarer remains of pleurodiran turtles (Dortokidae), birds and azhdarchid pterosaurs [17]. Of the latter, the new specimen presented here is the most complete European Maastrichtian azhdarchid found to date.

### Systematic Palaeontology

Pterosauria Kaup, 1834 [24]

Pterodactyloidea Plieninger, 1901 [25]

Azhdarchoidea Nessov, 1984 [26] (*sensu* Unwin [27]

Azhdarchidae Nessov, 1984 [26]

#### Remarks

EME VP 312 can be referred to the pterodactyloid clade Azhdarchidae due to the presence of the following characters in its cervical vertebrae: (1) extreme elongation of mid-cervicals; (2) reduced and/or vestigial neural spine; (3) tube-like cervical centra [26]–[29]. Similar to the Central Asian genus *Azhdarcho* but much younger in age and with the anatomical differences discussed below.

### *Eurazhdarcho langendorfensis* gen. et sp. nov

#### Holotype specimen

EME VP 312, a partial skeleton of medium-sized azhdarchid pterosaur (Figures 4–14).

**Figure 4:**
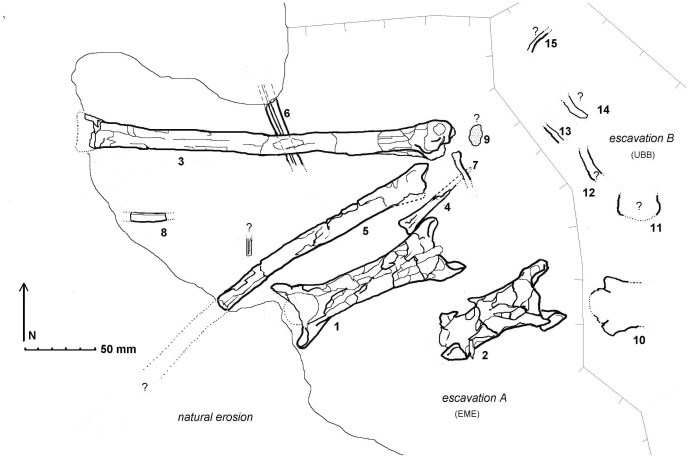
*Eurazhdarcho langendorfensis* (EME VP 312) *in situ* bone map. Numbered labels are as follows: cervical vertebrae (1, 2 and 10); third (#2) and fourth (#1) cervicals; right wing metacarpal four (# 3); incomplete right metacarpal three (# 4); proximal half of first right wing phalanx (# 5); portion of distal diaphysis of the second smaller wing phalanx (# 6); distal manual phalanx (# 7), and; several indeterminate fragmentary bones (# 8, 9, 11–15) some perhaps pertaining to additional manual phalanges and/or small metacarpals. As part of the associated skeletal elements of EME VP 312 (# 10–15) are now stored in the UBB collection (subsequent to collection; excavation B), closer examination for this project was not possible.

**Figure 5:**
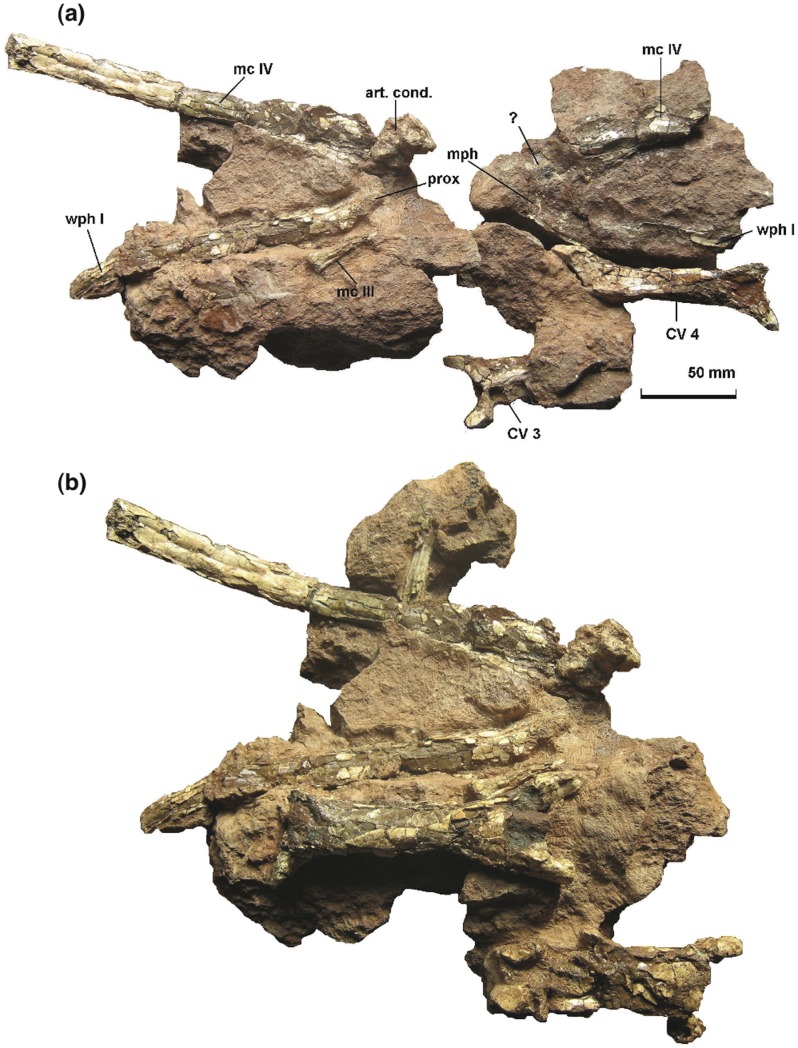
Preserved elements of *Eurazhdarcho langendorfensis* re-assembled as found in partial articulation. EME VP 312. (a) Slab and counter-slab with cervical vertebrae in dorsal view. (b) Specimen in original position with cervicals in ventral view. For scale in (b) see Table 1.

**Figure 6:**
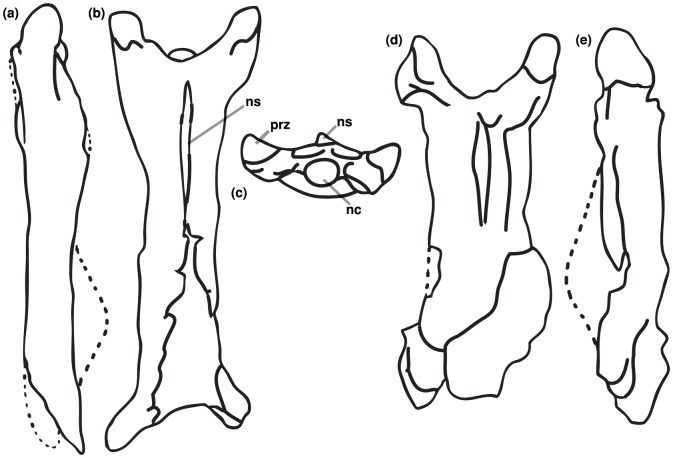
Line drawings of preserved *Eurazhdarcho langendorfensis* cervical vertebrae. EME VP 312. (a) Cervical four in lateral view. (b) Cervical four in dorsal view. (c) Cervical four in anterior view. (d) Cervical three in dorsal view. (h). Cervical three in lateral view. For scales see figures 7–9. Abbreviations: ns, neural spine; prz, prezygapophysis; nc, neural canal.

**Figure 7:**
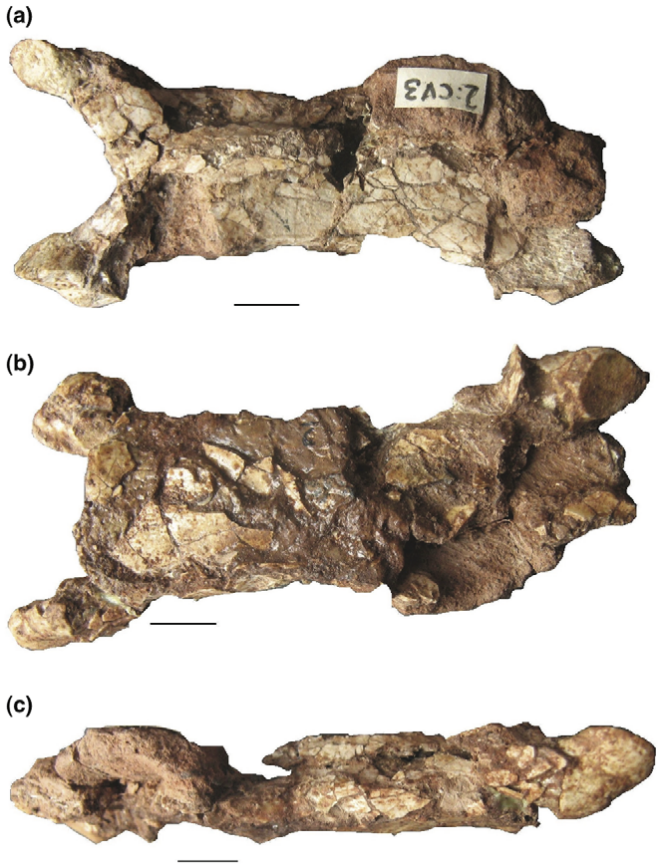
*Eurazhdarcho langendorfensis*, cervical vertebra three. EME VP 312/1. (a) Dorsal view. (b) Ventral view. (c) Lateral view. Scale bars are 10 mm.

**Figure 8:**
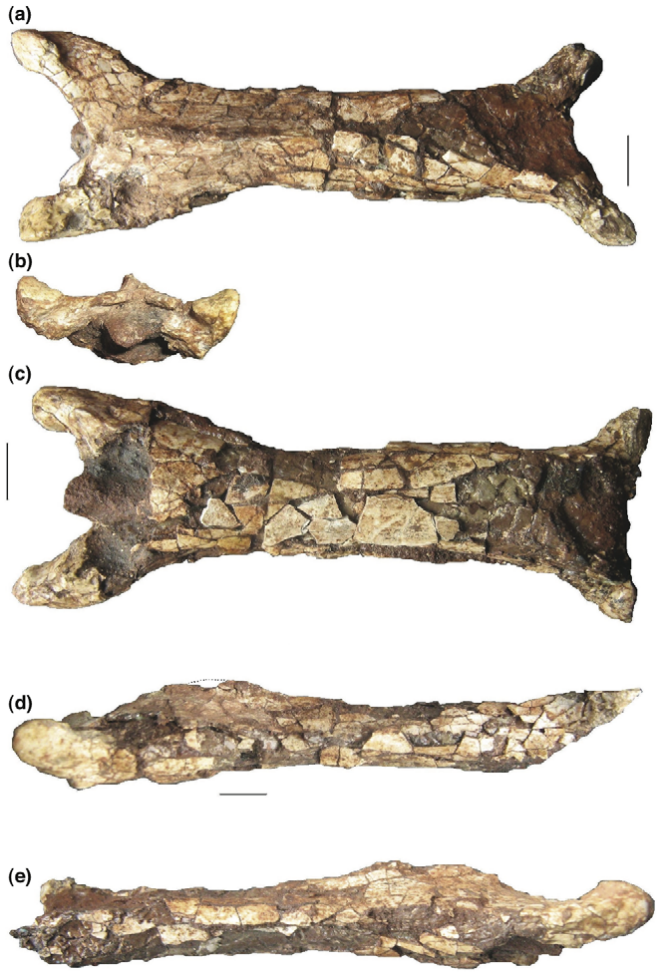
*Eurazhdarcho langendorfensis*, cervical vertebra four. EME VP 312/2. (a) Dorsal view. (b) Anterior view. (c) Ventral view. (d) Left lateral view. (e) Right lateral view. Scale bars are 10 mm.

**Figure 9:**
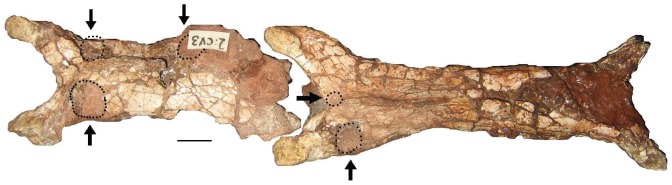
*Eurazhdarcho langendorfensis*, cervical vertebrae three and four in articulation. This view of EME VP 312/1 and 312/2 shows the presence of circular bitemarks (arrows) which we interpret as the scavenging activity of a ?small crocodylomorph. Scale bars are 10 mm.

**Figure 10:**
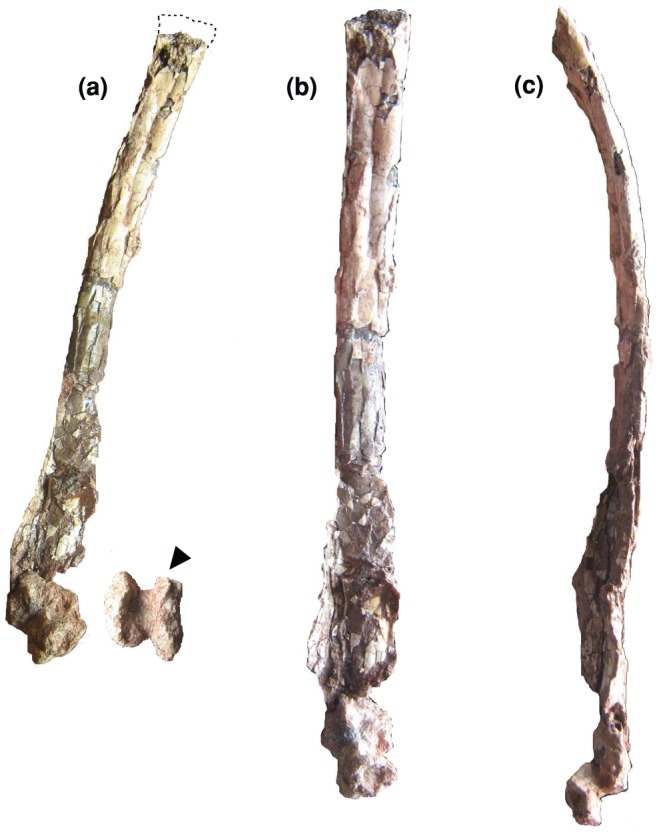
*Eurazhdarcho langendorfensis*, metacarpal four. EME VP 312/3. (a) Posterior and distal views. (b) Posterior view. (c) Lateral view. Total preserved length of this element is 236 mm (Table 1).

**Figure 11:**
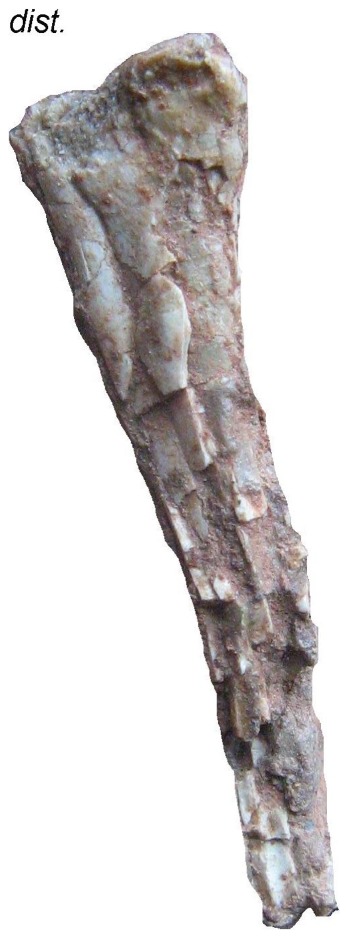
*Eurazhdarcho langendorfensis*, metacarpal three. EME VP 312/4 in ventral view. Total preserved length of this element is 39 mm (Table 1).

**Figure 12:**
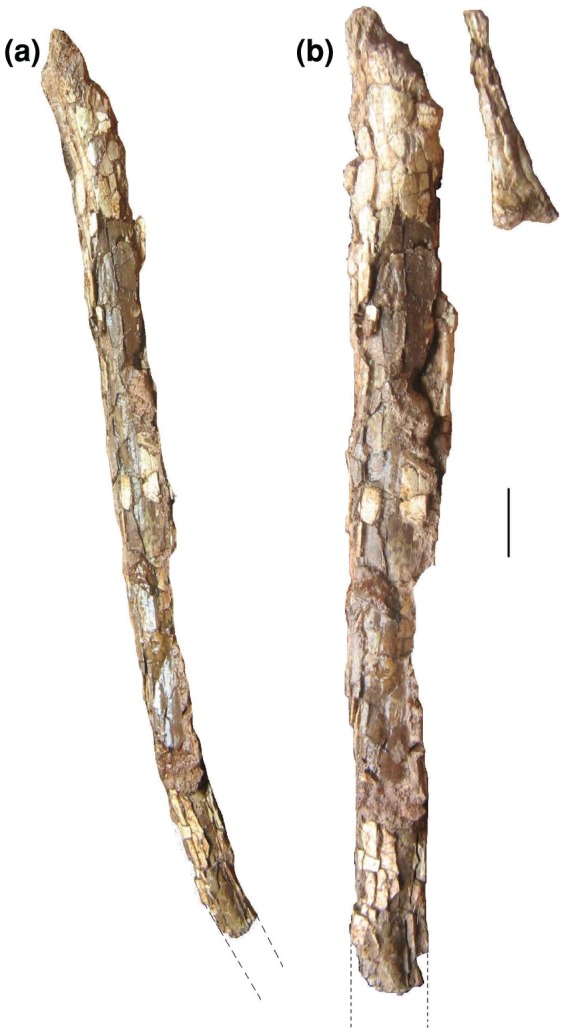
*Eurazhdarcho langendorfensis*, proximal portion of first wing phalanx. EME VP 312/5. (a) Lateral view. (b) Ventral view. Scale bar is 10 mm.

**Figure 13:**
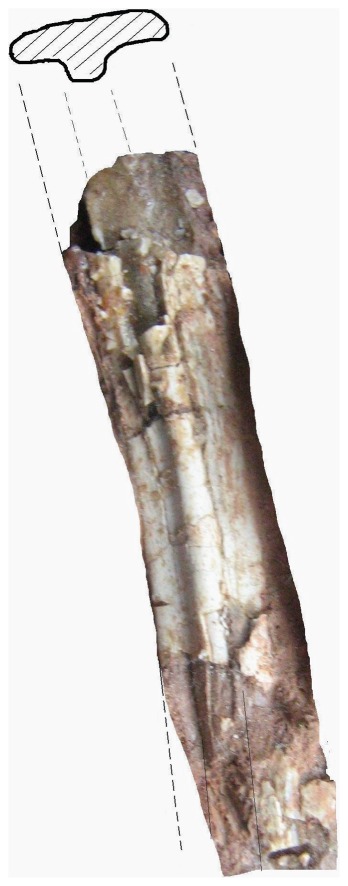
*Eurazhdarcho langendorfensis*, shaft fragment from second wing phalanx in ventral view. EME VP 312/6. Total preserved length of this element is 48 mm (Table 1).

**Figure 14:**
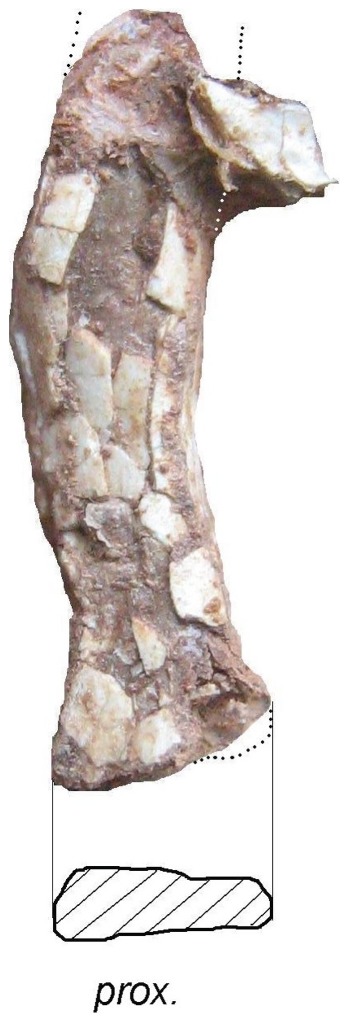
*Eurazhdarcho langendorfensis*, small manual phalanx in ventral view. EME VP 312/7. Total preserved length of this element is 19.5 mm (Table 1).

#### Etymology

*Eurazhdarcho* (urn:lsid:zoobank.org:pub:E1E2577C-85A2-4032-B0DD-FB4FCEAD82DB), combination of Europe and *Azhdarcho* (which comes from the Uzbek word *azhdarkho*, the name of a dragon in Uzbek mythology employed here to indicate European occurrence and close similarity with the Central Asiatic genus *Azhdarcho* Nessov, 1984; *langendorfensis* (urn:lsid:zoobank.org:act:1CE0CDD8-3DC7-4CED-B47B-E59D1B402936), derived from Langendorf, the ancient Transylvanian-German name of Lancrăm village, the Type locality.

#### Type locality

Lancrăm (Langendorf) near Sebeş, Alba district, Transylvania, Romania, Sebeş Formation, early Maastrichtian.

#### Diagnosis

The new taxon is diagnosed by the following proposed autapomorphies (which will, of course, be subject to test by later phylogenetic analysis): (1) Length of cervical three ca. 75% of cervical four (Table 1) (the same vertebral ratios in *Zhejiangopterus* and *Quetzalcoatlus* are closer to 60%; M. Witton, pers. comm. 2012); (2) Well-developed and elongated prezygapophyseal pedicles on cervical vertebrae that enclose an angle of 30 degrees with respect to the long axis; (3) Well-developed preexapophysis with an anteriorly oriented articular facet, separated from the external prezygapophyseal diapophysis via a deep sulcus;(4) Lateral pneumatic foramina small and situated lateroventrally with respect to the neural canal.

**Table 1:** Measurements of preserved elements of *Eurazhdarcho langendorfensis* (EME VP 312) (in mm).

No	element	Pres TL	Rec TL	CL	Ant W	Mid W	Post W	Min ER	Max ER
312/1	Cv4	116	∼120	88	39	19	39	3,0	6,1
312/2	Cv3	86,5	∼90	70	37	22	25	2,34	3,93

		Pres TL	Rec TL	Prox W	Prox D	Shaft W	shaft D	Dist W	Dist D
312/3	Mc IV	236	∼250	19	8,5	15	8,0 (11)	18	22
312/4	Mc III	39	∼60	-	-	4,5	3,5 (4)	13	6,1
312/5	Wph1	175	?	17	15	15	5,5 (10)	11	6.0
312/6	Wph2	48	?	-	-	8,5	4,0	-	-
312/7	Mph	19,5	∼23	7	4,5	4,5	4,0	-	-

Abbreviations: Cv = cervical vertebra; Mc = metacarpal; Wph = wing phalanx; Mph = manual phalanx; Pres TL = preserved total length; Rec TL = reconstructed total length; CL = corpus length; AntW = anterior width; MidW = minimum corpus width at mid-length; PostW = posterior width; MinER = minimum elongation ratio (PTL/AW); MaxER = maximum elongation ratio (PTL/MW); ProxW = prowimal width; ProxD = proximal depth; shaftW = shaft width; shaftD = shaft depth (in parentheses reconstructed shaft diameter); DistW = distal width; DistD = distal depth.

#### Differential diagnosis

Although relatively common in the Cretaceous fossil record, azhdarchid pterosaurs are typically represented by isolated remains. We compared EME VP 312 to other azhdarchid specimens for which overlapping elements are known. To date, most described specimens have been assumed to belong to a group of mid-sized and moderately long-necked azhdarchids, apparently the most common morphotype, in contrast to the more rarely recorded large to gigantic-sized very long-necked morph.

Compared to other known European Cretaceous fossils, EME VP 312 differs from the indeterminate azhdarchid cervicals described from Valencia [30] as these Spanish specimens are much larger in size, lack parasagittal carinae and have well-developed hypapophyses [30]). EME VP 312 is very different to the small azhdarchid cervical known from the lower Maastrichtian of Cruzy, Southern France [31]; this French specimen has short and robust prezygapophyseal pedicles, a diamond-shaped cross-section and a moderately concave triangular cotyle that has a ventral rim projected anteriorly to the hypapophysis [31]. Vertebrae of EME VP 312 can be distinguished from specimens (c.f. *Bakonydraco galaczy*) collected from the Santonian at Iharkut, Hungary, because of their smaller, ovoid cotylae and enlarged hypapophyses [32] (unpublished specimens in the Magyar Természettudományi Múzeum, Budapest, Hungary (MTM). We note that there are currently no overlapping elements between EME VP 312 and specimens referred to the giant-sized Haţeg azhdarchid *Hatzegopteryx thambema* [9].

In comparison with Asian azhdarchids, the similarly-sized *Azhdarcho lancicollis* Nessov, 1984 from the Turonian-Coniacian of Uzbekistan [33] nevertheless differs from EME VP 312 in its laterally constricted corpus and reduced hypapophyses that protrude beyond the pre-exapophyseal surface. *Aralazhdarcho bostobonensis* Averianov, 2007, from the upper Santonian-lower Campanian of Kazakhstan, is also different because it has extremely reduced lateral pneumatic foramina, larger cotylae, smaller prezygapophyses and a well-developed parasagittal carina [34]. *Volgadraco bogolubovi* Averianov et al., 2008 from the lower Campanian of the Saratov region (Russia) can be distinguished from EME VP 312 because of its comparatively short prezygapophyses, a large central pneumatic foramen above the neural canal, a short but well-expressed hypapophysis and a prominent parasagittal carina that connects the pre- and postzygapophyses.

EME VP 312 can be distinguished from the North African *Phosphatodraco mauritanicus* Pereda-Suberbiola et al., 2003, from the upper Maastrichtian of Ouled Abdoun because this Moroccan taxon has a vestigial neural spine that runs parallel to its long axis [35]. The much larger *Arambourgiania* is different because this taxon has a neural canal smaller than the lateral pneumatic foramina, posteriorly converging dorso-lateral parasagittal carinae, and small and robust prezygapophyseal pedicles [36].

### Description

EME VP 312 comprises closely associated and semi-articulated portions of the skeleton of a medium-sized azhdarchid pterosaur (Figures 4–14). The specimen consists of (at least) three anterior cervical vertebrae, a right wing metacarpal IV, an incomplete right metacarpal III, the proximal half of the first right wing phalanx, part of the distal diaphysis of the second smaller wing phalanx, a distal manual phalanx and several other fragmentary bones (Figures 4, 5). Some of these additional bones may represent manual phalanges and/or small metacarpals. Although not directly articulated, all the bones of EME VP 312 were found in close association: in the absence of any evidence to the contrary, we regard these elements as belonging to a single individual (Figures 4, 5). This is also consistent with bone proportions (Table 1).

The preservation of EME VP 312 is not exceptional and the cortical bone of several elements has been partially lost, leaving just impressions of internal bone molds. When actual bone is present, it is well-preserved (Figure 5) and in a similar condition to that reported in other pterosaurs (e.g., [37], [38]). Although some portions of individual elements have been crushed, they are not extremely flattened as is the case for many pterosaurs, notably similarly-aged specimens from the Niobrara Formation in Kansas, USA [37] and from the Jehol Group and older deposits of China (e.g., [39], [40]). Some elements of EME VP 312 are preserved in three dimensions, especially the cervicals, and this allows clear description of their morphology (Figure 5). They do not approach the condition, however, reported for pterosaurs from the well known Romualdo Formation (Santana Group) (Albian), Araripe Basin, Brazil (e.g., [41], [42]).

The remains of at least three cervical vertebrae (Figures 6–8) are identified here as part of EME VP 312. A fourth fragmentary bone (? in Figure 5) might also turn out to be a cervical element, but it consists of only its middle portion and is too compressed to allow a definitive interpretation.

The best-preserved cervical vertebra of EME VP 312/1 is an elongate and gracile element that we interpret as cervical four (Figure 6). It has expanded pre- and postzygapophyseal areas and is relatively uncrushed, preserving most of the cortical bone surface and, anteriorly, the internal mold of the neural canal. It consists of the partial anterior articular region, most of the corpus (cortex slightly damaged anteroventrally, posterodorsally and caudoventrally) and both (slightly damaged) postzygapophyses (Figure 6). The preserved minimum elongation ratio of this element is ∼3,0 while the maximum elongation ratio is ∼6,0 (Table 1). EME VP 312/1 also has well-developed and elongated prezygapophyseal pedicles that enclose an angle of 30 degrees with respect to the long axis, comparatively large preexapophyses with anteriomedially oriented articular facets that are separated from the prezygapophyseal tubercles by deep and wide ventral sulci. This latter feature suggests the remnant of an extremely reduced cervical rib similar to that observed in some other azhdarchids [43]. There are also large anteromedially oriented semicircular to oval prezygapophyseal facets with anteroposteriorly convex and lateromedially concave articular surfaces (Figure 6). The neural canal of EME VP 312/1 is preserved as a prominent internal mold positioned at mid-height between the preexapophyses. The corpus of this vertebrae is elongate and with a dorsoventrally sub-oval cross-section and parallel lateral margins along its length (Figure 6). Anterodorsally along the spine, the corpus is slightly concave and becomes convex posteriorly; ventrally, the surface of the corpus is slightly convex and lacks hypaphophyses. There is a low and blade-like neural spine (Figure 6) (except anteriorly where this is broken) that is relatively elevated and divided into anterior and posterior ridges separated with an elongate low gap. The anterior section of this spine, slightly truncated posteriorly, comprises 30 percent of the whole vertebral length.

Another cervical vertebra (EME VP 312/2) is compressed dorsoventrally and is distorted on its right side. We interpret this element as cervical three (Figure 7): it consists of the posterior portion of the centrum (the posterior articular region is damaged), the right postzygapophysis, a small part of the left postzygapophyseal facet and partial condyle and most of the corpus with a low dorsal neural spine. The element is moderately elongated with a preserved centrum length of 86.5 mm (thus, total estimated length 90 mm) The maximum width of the central portion of this vertebra is 22 mm, giving a maximum elongation ratio close to 4.0(Table 1). The centrum of EME VP 312/2 is hollow and has an average bone thickness of ca. 1 mm. It is slightly higher than wide and lacks lateral pneumatic foramina (Figure 7). The dorsal surface of this element is slightly convex and gives the whole centrum a tube-like outline while a small groove is present on the middle portion of the dorsal rim of the cotyle, dorsal to the neural channel. Poor preservation means that it is not possible to determine the presence of a lateral pneumatic foramen on either side of the neural canal, as is present in some other azhdarchid [26], [43], [44] and tapejarid [45] azhdarchoids.

The anterior articular region of this vertebra, although slightly crushed (2.9 times wider than high), nevertheless bears some distinctive features (Figure 7): well-developed and elongated prezygapophyseal pedicles that enclose an angle of 35 degrees with respect to the long axis; a relatively wide and low cotyle with a small lateral accessory articular surface dorsally and below the neural canal; a well-developed preexapophysis with an anteriorly oriented articular facet, separated from the external prezygapophyseal diapophysis via a deep sulcus; comparatively large anteroposteriorly elongated and slightly convex oval prezygapophyseal facets that are anteromedially oriented. The relatively small neural canal opening of EME VP 312/2 is positioned immediately under the sharp and well-developed interzygapophyseal ridge, while the lateral pneumatic foramina are smaller and are situated lateroventrally (Figure 7). At interprezygapophyseal level, the neural spine and hypapophyses dorsally and ventrally are completely reduced, thus the anterior ventral surface of the corpus is flat and slightly convex (Figure 7).

The prezygapophyseal articulations between EME VP 312/1 and 312/2 match perfectly (Figure 8) and further corroborate our anatomical identifications. Based on the morphology of these articular surfaces, it is also noteworthy that little or no lateral and limited vertical movement would have been possible at the junction between cervicals 3 and 4 (no more than 20 to 30 degrees). However, the shape of the prezygapophyseal articulation on the third cervical indicates greater mobility at the atlas-axis/cervical 3 junction, up to 45 degrees vertically. These are in agreement with similar values calculated for a mid-sized azhdarchid (?*Azhdarcho*) from the Cenomanian-Santonian of Burkhant, Mongolia [46].

The third identifiable cervical vertebrae that comprises EME VP 312 (labelled 10 on Figure 4) is now housed in the UBB collection and is inaccessible to us. It consists of the posterior portion of a centrum with left postzygapophysis. All three vertebrae comprising EME VP 312 were mostly likely articulated and belonging to the anterior part of the neck: most likely the atlas-axis and cervicals three and four (Figure 4).

In comparison with other described azhdarchid taxa, the preserved mid-cervical elements of EME VP 312 are extremely elongate and bear less reduced neural spines, especially cervical three (Figures 6–8). The vertebrae of EME VP 312 also have low neural arches that are confluent with their respective centra: superficially, they form tubes with reduced, or absent, cervical ribs. Of these characters, the first three are proposed synapomorphies of Azhdarchidae [27], [29] while the absence of marked pneumatopores on the cervical centra may represent an additional autapomorphy (or intraspecifically variable feature) of this new medium-sized Romanian taxon (see Discussion). The cervicals of EME VP 312 are also similar to those from the Upper Cretaceous (Santonian) Csehbánya Formation that outcrops in the Bakony Mountains, Hungary. Several elements (some still unpublished, Attila Ösi pers. comm. 2011) were found associated with the Hungarian azhdarchid *Bakonydraco galaczi* and might be referable to this species [32]. For example, EME VP 312/1 cervical four has a length/minimum width ratio that is very similar to MTM Gyn/448, also regarded as a likely fourth cervical element (Attila Ösi pers. comm. 2011). It is also noteworthy that in MTM Gyn/448 the prezygapophyseal pedicels are shorter, much more robust and have a different enclosure angle with respect to their long axes than is the case in EME VP 312.

The cervicals of EME VP 312 also share the presence of well-developed hypapophyses and parasagittal carinae with described material of *Aralazhdarcho bostobonensis* from the late Santonian-early Campanian of Kazakhstan [34]. The Romanian specimen, however, differs in the possession of extremely reduced lateral pneumatic foramina, a larger cotyle and smaller prezygapophyses. A low neural spine is also seen in the North American azhdarchid *Montanazhdarcho minor* [48], [49] from the Campanian Two Medicine Formation, Montana, where the spine is even shorter and lower than in EME VP 312.

An additional, similar-sized azhdarchid, *Volgadraco bogolubovi* [50], is known from the lower Campanian of the Saratov region, Russia. This species was described on the basis of scattered remains that apparently belong to several individuals, including a third cervical. If this cervical of *Volgadraco* is correctly identified, it differs from EME VP 312 in being comparatively shorter, in possessing an anteriorly high neural arch, a large central pneumatic foramen located above the neural canal, small pneumatic foramina on the lateral side of its corpus (although this may reflect individual variation), a short but prominent hypapophysis, and a prominent parasagittal carina connecting the pre- and postzygapophyses. However, we suggest that the length of this element in *Volgadraco*, combined both with the presence of a dorsal pneumatic foramen located above the neural canal and a well-expressed hypapophysis, indicate that this is a more posterior cervical vertebra than described (see, for example, the condition in *Azhdarcho*).

EME VP 312 also comprises at least 6 wing-bones (312/3-8) but, due to their fragility, most of these are heavily crushed and/or distorted and fragmented (Figures 5, 9–11). Other elements (numbered 12–15 in Figure 4) may also represent small metacarpals and/or phalanges.

Of these preserved elongate elements, EME VP 312/3 is the longest (Table 1) and best preserved (Figure 10). We interpret this element as the fourth right wing metacarpal (Figure 10): it is almost complete (only the proximal epiphysis is missing), very elongate and gracile, and most of its cortex is preserved, though heavily crushed antero-posteriorly. The distal articular region is also distorted and shifted about 45–50 degrees laterally. The proximal region of the shaft close to the articulation in EME VP 312/3 is subrectangular, less compressed and has a longitudinal groove and a small subcircular depression (Figure 10). The diaphysis is straight but compressed (reconstructed midshaft diameter ∼11 mm), and narrows distally. The cortex has an average thickness of 1 mm, with smooth internal surface on the proximal 2/3, and has well-expressed trabecular internal structure toward its distal end. The intercondylar groove on the distal end is deep, without a rounded median crest or ridge: a similar condition is seen in *Azhdarcho* and *Montanazhdarcho* [48], [49].The distal-most part of the shaft is completely flattened and has two more-or-less circular (possible) puncture marks.

Discovered lying parallel to the first wing phalanx, a smaller long bone is interpreted here as the third metacarpal (EME VP 312/4) (Figures 4, 11). Only about two-thirds of this bone remains (Table 1): it has a diaphysis with a subtriangular to suboval cross-section that narrows proximally and becomes flared distally. The distal epiphysis is wide and triangular in shape and has a slightly convex articular surface that is partially damaged.

The second longest bone preserved as part of this specimen (EME VP 312/5) we interpret to be the first phalanx of the fourth wing finger (Figure 12) as it is preserved in semi-articulation with metacarpal four (Figure 4). About two-thirds of this bone is preserved (the distal region is missing). It is dorsoventrally crushed and most of the external cortical bone is missing (Figure 12). The proximal articular region of EME VP 312/5 is damaged, but a proximally prominent triangular structure, similar to an extensor tendinal process, is preserved (Figure 12). If this interpretation is correct, the specimen represents a relatively mature individual [51], [52]: Complete ossification and fusion of this region is seen in later ontogenetic stages [51], [52]. The articular surface of EME VP 312/5 is also badly preserved but the ventral articular cotyle is better expressed: the diaphysis is dorsoventrally compressed and hollow. The average bone thickness at the diaphysis is ca. 1 mm but reaches a maximum of ca. 3 mm proximally. This bone also has an oval transverse section with the middle part of its diaphysis narrowing toward its distal end (Figure 12).

EME VP 312/6 is a small shaft fragment (length, 52 mm; average width, 8 mm) that we interpret as being from part of the mid-distal diaphysis of a smaller phalanx, most likely the second phalanx of wing finger four (Figure 13). This element has the typical (inverted) “T” shaped ventral transverse cross section that is seen in azhdarchid [47], [48] and tapejarid (A. Kellner, pers. obs. 2011) pterosaurs. The strong ventral ridge of this element, placed centrally along the shaft and becoming slightly shifted posteriorly towards the proximal end, is reminiscent of the condition seen in *Azhdarcho lancicollis* [33], [50].

EME VP 312/7 is a small bone that was found in close association with the metacarpals (length, 15 mm; proximal depth, 5 mm): it is interpreted here as a distal manual phalanx (Figure 14) that has a subrectangular, widened proximal epiphysis and a slightly curved, distally crushed diaphysis with an oval cross section. Proximally, the articular surface is concave but damaged: although apparently lacking a lateral or latero-ventral pneumatic foramen, this bone is similar to the distal manual phalanges of *Azhdarcho* from the Turonian of Uzbekistan [33], particularly in the size and shape of its diaphysis.

EME VP 312/8, is a small elongate bone fragment, tentatively interpreted here to be the distal section of the terminal wing phalanx. This small bone has a low triangular-to-oval cross section (it lacks a ventral ridge) and a comparatively thick cortex (ca. 1.0 mm). Similar bone fragments have been described from the Campanian-Maastrichtian of Campagne-sur-Aude in southern France [51], [54] and from the Santonian of Iharkut, Hungary [47]. However, in *Azhdarcho lancicollis*, the fourth wing phalanx is rod-like, has a triangular cross-section and, distally, lacks a ventral ridge [33].

### Estimated Wingspan

**Figure 15:**
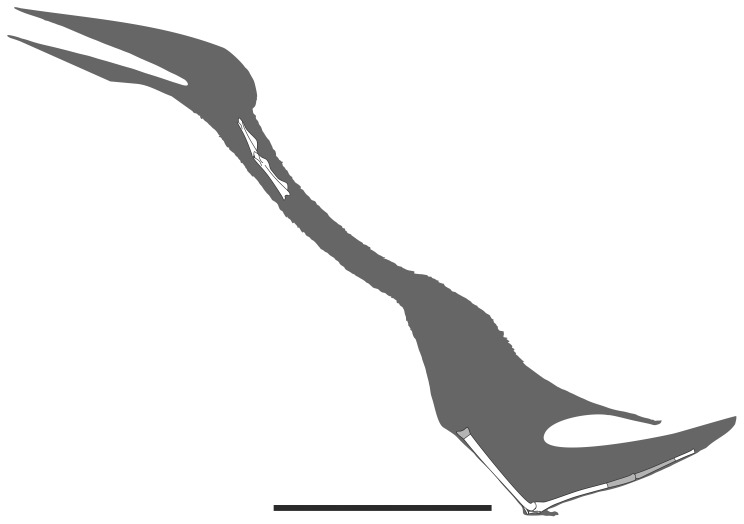
Full body reconstruction of *Eurazhdarcho langendorfensis*. Preserved skeletal elements are in white. Scale bar is 500 mm. Image courtesy of Mark Witton.

None of the wing bones that comprise EME VP 312 are complete enough to allow us to provide an accurate estimate for the wing-proportions and wing-span of this new pterosaur. However, one wing bone is relatively complete: metacarpal four (missing only its proximal articulation). The reconstructed original length of this element is 250 mm with an original mid-shaft diameter of 11 mm (Table 1). Compared to the wing-bones and cervicals of the largest (adult) and most complete specimen of *Zhejiangopterus* [53], [55], it is of note that the length of cervical four is roughly the same as in EME VP 312 (Table 1; ca. 114 mm), although our estimate for the original length of metacarpal four is substantially less (250 vs 336 mm). If correct, this comparison would suggest a smaller wing-span for EME VP 312, no more than 3 m (cf. 3.5 m for the largest known individual of *Zhejiangopterus*) [55]. A full-body reconstruction of *E. langendorfensis* is given in figure 15.

## Discussion

### Taphonomic Implications

The EME VP 312 bone map (Figure 4), combined with data from local sedimentology (Figure 3), suggests that the carcass of *Eurazhdarcho langendorfensis* was probably transported in a low energy current during a waning flood (perhaps a monsoonal event). The specimen was then deposited on its back on mud at the proximal part of a floodplain. Bone preservation suggests that at least some parts of the skeleton were subaerially exposed for an unknown period of time, since they are disarticulated and fragmented yet do not exhibit signs of hydrodynamic reworking. A few unusual breaks and subcircular puncture marks are present on both mid-cervicals and on the distal part of the large metacarpal (Figure 9): this damage may be due to scavenging activity, and it is possible that this behaviour contributed to the disarticulation. Two unequal circular puncture marks are present at the base of the left prezygapophyseal pedicle and anterior to the neural spine on the concave surface of cervical four (EME VP 312/1); more extensive ventral breakage is present on the convex surface. The morphology and distribution of these marks (Figure 9) suggest crushing by tapering objects, and they are probably bite-marks made by a conical-toothed scavenger. A similarly scavenged azhdarchid specimen was reported from the Campanian of Alberta in Canada [56]. In this case, an embedded tooth tip and well defined, linear bite marks identified the scavenger as the dromeosaurid *Saurornitholestes*. Bite marks were also reported on a pteranodontoid metacarpal from the Albian Toolebuc Formation of Australia, but no further indication of the scavenger could be found [57]. The bite marks on EME VP 312 are morphologically quite different to those inferred to be produced by dromeosaurids and are more similar to crocodyliform-style bite marks frequently recorded in the Sebeş Formation, particularly at the Oarda de Jos locality [23]. Because of our unsuccessful subsequent excavations at the quarry site, we suspect that most of the remaining skeleton was either recently eroded or (more likely) dispersed by scavengers.

Sedimentological and taphonomic evidence suggests a general attritional and trampled taphofacies of largely isolated and evenly distributed bone fragments and splinters. Commonly, other preserved (mostly fragmentary) bones suffered long term subaerial biodegradation, disarticulation and prefossilisation weathering (stage 3–5), with occasional insect-related surficial modifications being present as well. Most insect marks on the bones may be assigned to coleopterans (Dermestidae?) and isopterans (termites), generally pointing to long-term subaerial exposure, low water table, a sparsely vegetated environment and relatively dry conditions [13], [58]. In some cases, episodic flood-related fine sediment input covered the sometimes transported, partially decomposed or scavenged carcasses. Two such levels have been identified, the lower one (restricted to several square metres) containing a dromaeosaurid theropod (*Balaur bondoc*) partial skeleton [16], [17], whereas the upper one (more exposed) has so far yielded the partial skeletons of this pterosaur, a semi-terrestrial stem-turtle (*Kallokibotion bajazidi*), a hadrosaur (*Telmatosaurus*), a titanosaur (cf. *Magyarosaurus*) and/or other relatively complete or associated fragile bones, including a multituberculate mammal. In certain areas, around and associated to the dispersed skeletons, crocodylomorph shed teeth and well preserved coprolites are common. No doubt, and taking into consideration the very limited exposure (less than 200 m^2^), Glod/B is the most productive and promising vertebrate-site in the whole Sebeş area.

### Distribution and Paleoecology

Prior to our description of *Eurazhdarcho langendorfensis*, the only specimens noted in the literature are the shaft of a wing phalanx from the top of the Bozeş Formation and an extremely large cervical vertebra from the Râpa Roşie at the Sebeş area (Sebeş Formation) [1], [13], [14]. A number of other elements are known and their study represents ongoing aspects of our research in Romania.

The discovery of azhdarchid material (*Eurazhdarcho langendorfensis*) considered morphologically similar to *Azhdarcho* is noteworthy; we note, however, that *Azhdarcho*-like azhdarchids have has previously been reported from Europe [59]. Buffetaut [59] identified a series of disassociated remains from the Upper Cretaceous of the Lãno locality in Spanish Basque Country – including a probable lower jaw fragment, cervical vertebrae, a notarium, sacrum, femur and some poorly preserved wing bones – as cf. *Azhdarcho* sp. ([59] p. 290). This referral was made because the Lãno cervical vertebrae have a “blunt and weakly marked” hypapophysis with adjacent “weak, posteriorly convergent ridges on both sides” and prominent sulci lateral to the ridges and positioned anteriorly, close to the prezygapophyses ([59] p. 290). An identification as cf. *Azhdarcho* was favoured since the only other azhdarchids with described cervical vertebrae known at the time (*Quetzalcoatlus* and *Arambourgiania*) were either much larger or (in the case of *Arambourgiania*) different in lacking the lateral ridges and sulci. Buffetaut [59] noted that this referral was provisional. Additional azhdarchid material from La Solana in the Tous area of Valencia, Spain, has been suggested to have affinities with the Lãno material [60] despite possessing a more prominent hypapophysis and apparently lacking lateral sulci. Note, however, that there is certainly an element of subjectivity in determining whether a hypapophysis is prominent or not.

Knowledge of azhdarchids has been augmented in recent years by revisions of existing collections [61] and descriptions of new material and new taxa from eastern Europe (e.g., [9], [32]), Russia (e.g., [34], [50]), North Africa (e.g., [35]), North America (e.g., [49]) and Asia [62], including some fragmentary specimens tentatively referred to the group [63]. Some of this material indicates that the characters used to identify the Lãno material as cf. *Azhdarcho* are possibly widespread within the group: the hypapophysis, for example, is weakly expressed in *Bakonydraco galaczi* [40] and lateral sulci and associated ridges are present in *Phosphatodraco mauritanicus* [34], *Bakonydraco galaczi* [47] and in additional North African [49] specimens. The Lãno azhdarchid material does not, therefore, necessarily indicate the presence of an *Azhdarcho*-like taxon since the characters initially used to support this assignment have become obsolescent characters.

The discovery of *Eurazhdarcho langendorfensis* within the Transylvanian Basin also augments out knowledge of azhdarchid palaeoecology and behaviour. As is the case with many other azhdarchid remains known from elsewhere [60], *E. langendorfensis* is associated with an assemblage of dinosaurs and other terrestrial animals. This seems to further corroborate suggestions that azhdarchids were animals of continental environments such as woodlands, riverplains and scrublands; they were not routinely marine or strongly associated with coastal habitats as proposed by some authors. Witton and Naish [60] argued that azhdarchids were ‘terrestrial stalkers’ that, while evidently capable of crossing large distances via dynamic soaring, foraged quadrupedally in diverse environments for small animal prey in additional, hypothetically, to carrion and edible plant material.

**Figure 16:**
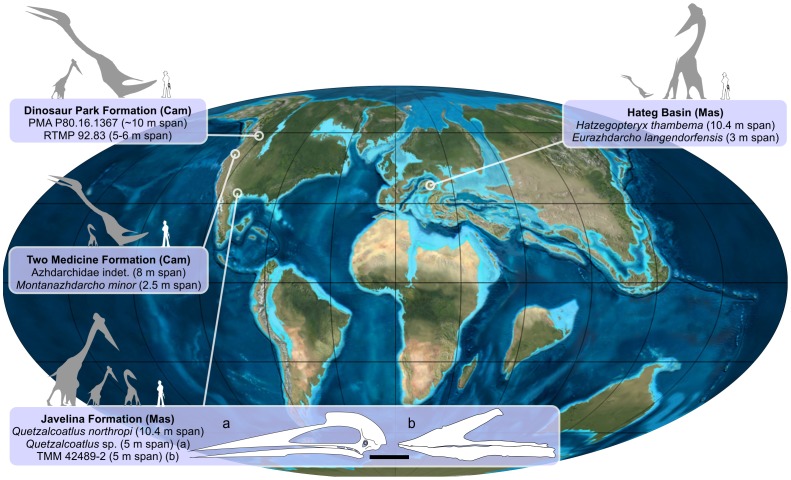
Map to show the global distribution of faunas containing small-medium and giant-sized azhdarchids, evidence for niche partioning. Image rendering courtesy of Mark Witton; map imagery by kind permission of Ron Blakey, Colorado Plateau Geosystems, Inc.

Also of palaeoecological interest is the fact that *E. langendorfensis* – representing a mid-sized azhdarchid apparently similar to *Azhdarcho* – is approximately contemporaneous with the gargantuan azhdarchid material known from the Sebeş Formation at the Râpa Roşie (Red Cliff) locality. The Râpa Roşie material (including a cervical vertebra, syncarpal and scapulocoracoid) represents one of the largest, in fact likely *the* largest, azhdarchoid yet discovered [1], [13], [14] and is currently under study by our team. It remains unknown whether EME VP 312 and the Râpa Roşie material were truly contemporaneous and whether the azhdarchids concerned might have overlapped in habitat and ecological preference. However, in the absence of further information, it is reasonable to assume that both these Maastrichtian animals did indeed overlap in time and space, especially given the assumed ability of large and giant pterosaurs to cover distance with relative ease [10] (though note that both fossils are from distinct sedimentary basins). There are presently no reasons to assume that both represent growth stages of the same taxon (EME VP 312 is most likely an adult, not a juvenile of the taxon represented by the Râpa Roşie specimens); therefore, we conclude that both medium-sized and gigantic azhdarchids co-existed in the Romanian Cretaceous and were presumably able to avoid competition via niche partitioning. This situation is actually characteristic of a number of other Cretaceous pterosaur faunas worldwide; jaw morphologies from the Cretaceous Javelina Formation are evidence for niche partitioning in azhdarchid feeding styles at different body sizes (Figure 16). Note that confirmation of our hypothesis requires the discovery of material demonstrating distinct feeding styles or ecological preferences and these comments are preliminary.

*Eurazhdarcho langendorfensis* is one of the most significant azhdarchid specimens known from Europe. Furthermore, it is of special interest in being about 20 million years younger than apparently similar forms from Central Asia (e.g., [34]) and Mongolia [46]. Because most azhdarchid specimens consist of isolated or scattered elements that are often poorly preserved, incomplete and/or crushed, evaluation of taxonomic diversity, particularly in the Upper Cretaceous European record, has proved problematic. Indeed, most described specimens have been allocated to Azhdarchidae on the basis of their elongate, sub-cylindrical cervical vertebrae. Mid-sized cervical vertebrae of this type are widely distributed enough to suggest that moderately long-necked, medium-sized azhdarchids were distributed virtually globally during the Late Cretaceous. Indeed, this vertebral form seems the most common morphotype, in contrast to the more rarely recorded large or gigantic morphotypes.

## Methods

### Nomenclatural Acts

The electronic edition of this article conforms to the requirements of the amended International Code of Zoological Nomenclature, and hence the new names contained herein are available under that Code from the electronic edition of this article. This published work and the nomenclatural acts it contains have been registered in ZooBank, the online registration system for the ICZN. The ZooBank LSIDs (Life Science Identifiers) can be resolved and the associated information viewed through any standard web browser by appending the LSID to the prefix “http://zoobank.org/”. The LSID for this publication is: urn:lsid:zoobank.org:pub:8D31134D-25B4-4357-9FE8-C74DC51735BC. The electronic edition of this work was published in a journal with an ISSN, and has been archived and is available from the following digital repositories: PubMed Central, LOCKSS.
